# Understorey to canopy vertebrate fauna of a lowland evergreen forest in Mt. Makiling Forest Reserve, Philippines

**DOI:** 10.3897/BDJ.8.e56999

**Published:** 2020-11-03

**Authors:** Juan Carlos T. Gonzalez, Anna Pauline O. de Guia, Judeline C. Dimalibot, Khryss V. Pantua, Whizvir O. Gustilo, Nathaniel C. Bantayan

**Affiliations:** 1 Museum of Natural History, University of the Philippines Los Baños, Los Baños, Laguna, Philippines Museum of Natural History, University of the Philippines Los Baños Los Baños, Laguna Philippines; 2 Animal Biology Division, Institute of Biological Sciences, College of Arts and Sciences, U.P. Los Baños, Los Baños, Laguna, Philippines Animal Biology Division, Institute of Biological Sciences, College of Arts and Sciences, U.P. Los Baños Los Baños, Laguna Philippines; 3 Institute of Renewable Natural Resources, College of Forestry and Natural Resources, U.P. Los Baños, Los Baños, Laguna, Philippines Institute of Renewable Natural Resources, College of Forestry and Natural Resources, U.P. Los Baños Los Baños, Laguna Philippines

**Keywords:** vertical stratification, tropical rainforest, wildlife, species richness

## Abstract

We examined the vertical stratification of forest wildlife, from the ground up to the canopy layer, within a 2-hectare permanent plot of lowland evergreen rainforest on the Mt. Makiling Forest Reserve. Our aim was to determine the species richness of the different forest layers and evaluate their ecosystem services. Understorey, sub-canopy and canopy sampling were conducted during July 2016, March to April 2017 and February to March 2018, respectively. We were able to record a total of 68 species, consisting of 11 amphibians, 15 reptiles, 25 birds and 17 mammals. Increasing species richness with increasing vertical stratification was observed for both reptiles and mammals. For birds, the peak richness was observed in the sub-canopy and then decreased in the canopy. A decreasing trend was observed with amphibians wherein the peak species richness was observed in the understorey. Increasing vertical stratification influenced vertical habitat use and species richness. For the similarity index, the same pattern was observed for all species groups. Highest similarity was observed between the sub-canopy and the canopy and the least similarity was observed between the understorey and canopy. These results indicate that the understorey and the canopy host different species groups, thus, sampling of the understorey alone, often done in biodiversity surveys, may lead to the underestimation of species richness in an area.

## Introduction

Tropical forests exhibit heterogeneity and high vertical complexity brought about by varied tree heights which help maintain and increase species diversity (Vieira and Monteiro-Filho 2003). They can be vertically divided into distinct layers or strata, characterised by differing levels of water, sunlight and air circulation, but are interdependent (National Geographic Society 2020). Vertical stratification in tropical forests can be separated into three to four forest heights from ground level (<1.5 m), namely the understorey (1.5-2 m), sub-canopy (2-6 m), canopy (6-15 m) and emergent layers (Vieira and Monteiro-Filho 2003, Adams et al. 2009, Anonymous 2019). This vertical stratification of habitat layers of plant and animal communities represent a basic concept in forest ecology, which has not been well examined for patterns and often defined differently (Smith 1973, Parker and Brown 2000). Drivers of canopy stratification were found to be significant for forests with varied leaf and top heights, clustered leaf heights and those harbouring different life forms at different heights (Parker and Brown 2000). The degree of canopy stratification and woody species diversity are influenced by latitudinal thermal gradients from subtropics to tropics and this multi-layering structure helps maintain high species diversity (Feroz et al. 2015). Thus, a stratified structure is more evident in tropical than in temperate forests and this layering of vegetation affects the distribution of food resources, microclimate and faunal composition (Smith 1973).

The forest canopy refers to the upper layer or habitat zone, formed by interspersed crowns of mature trees and includes other biological organisms, such as epiphytes, bryophytes, lianas and mistletoes forming a community of associated flora and fauna (Parker 1995). For a single tree, the term “canopy” is used to refer to the extent of the outer layer of leaves of an individual tree forming its crown cover. Together, these crowns form the canopy, wherein the lower branches of the tallest trees are often in contact with the leaves and branches of adjacent trees, allowing horizontal access. Towering above the contiguous main canopy are tall crowns of scattered emergent trees, also called overstorey. Within the dense ceiling of the canopy, there is an abundance of leaves and fruits that provide food for herbivores which, in turn, are predated upon by carnivores. The middle sub-canopy normally lies 2-5 m above the ground, just below the first branches of the canopy and consists mainly of small juvenile trees, palms and woody species adapted to low light filtered by dense foliage. The understorey includes shrub and herb layers of undergrowth, consisting mostly of young saplings, shade-tolerant herbaceous shrubs and ferns slowly reaching for light from the forest floor.

Mt. Makiling is considered a low mountain with its highest peak of only 1090 m a.s.l. Despite this, the mountain uniquely consists of various habitat types, such as lowland evergreen, montane and mossy rainforests. The Mt. Makiling Forest Reserve (MMFR), due to its accessibility and historical association with the University of the Philippines Los Baños (UPLB) as a field laboratory, is one of the most studied of the ASEAN Heritage Parks (AHP) in the Philippines. Assessment of its vertebrate fauna has been noted since the early part of the 20th century (Taylor 1922), but only fully documented in the later part, especially for birds (Alviola 1977, Miranda 1987, Mendoza 1987), bats (Ingle 1992) and herpetofauna (Custodio 1986, Alcala et al. 1997, Gonzalez and Dans 1994). A more comprehensive wildlife assessment across elevational and habitat gradients was undertaken by Gonzalez and Dans in 1998 (Sajise et al. 2005), which was followed by successive studies on habitat gradients and forest stratification, such as on understorey birds and small mammals (Gonzalez 2005, Gonzalez 2008, de Guia et al. 2011), bats (Alviola 2008, Sedlock 2001, Sedlock 2002) and montane wildlife (Abraham et al. 2010). Despite the long history of biodiversity assessment, none of the studies effectively assessed the wildlife found in the upper forest strata, from sub-canopy to emergent layer. To some extent, sampling for insectivorous bats in the forest canopy has been included (Sedlock 2001, Sedlock 2002),thus showcasing the strong potential for conducting “forest canopy studies” at MMFR. Despite these comprehensive studies on the faunal diversity of MMFR, some areas of the Park remain to be fully explored – for example, the ecology and natural history of vertebrate fauna found in different forest strata, especially through the upper forest canopy and emergent layer.

Vertical stratification is the most significant factor in the diversification of forest habitats in Mt. Makiling, as it provides potential niches by driving species to adapt for aerial and arboreal habits (Gonzalez and Dans 1997). The tropical lowland evergreen rainforest contains most of the layers amongst those forest types categorised by Fernando et al. (2008) and amongst the four types of vegetation recognised in Mt. Makiling (Lapitan et al. 2013). This multi-tiered structure of lowland forests at established monitoring plots within MMFR was the focus of the Forest Canopy Observation, Positioning and Investigation (Forest CANOPI) Program, an inter-disciplinary research which covered various aspects of canopy science, from mapping crown structure to assessing biodiversity (fauna, flora and microbial diversity). Our study was part of the programme centred on understanding tetrapod diversity across vertical layers and horizontal zones within a lowland evergreen rainforest. In particular, this paper aimed to determine species richness amongst terrestrial vertebrate fauna (amphibians, reptiles, birds and mammals) distributed across three lowland forest strata on Mt. Makiling (understorey, sub-canopy and canopy) and to be able to compare composition of tetrapod fauna, based on diet and guilds to evaluate their equivalent contributions in providing ecosystem services.

## Methodology

### Site Description

**Figure 1.**
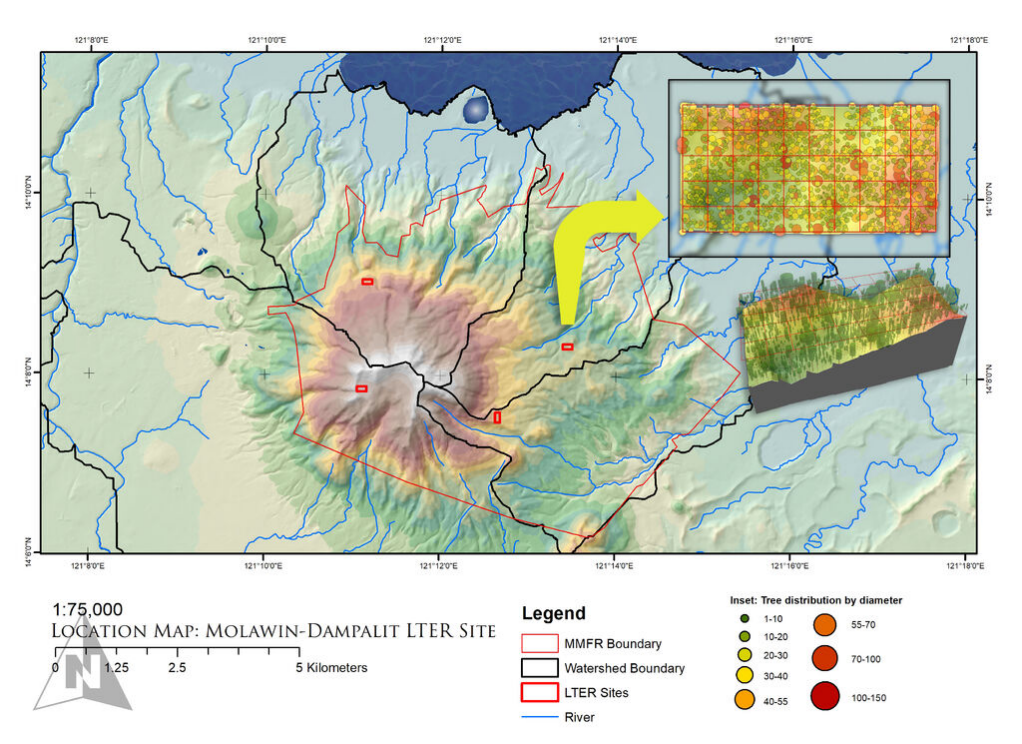
Location map of the Molawin-Dampalit Permanent Plot inside Mt. Makiling Forest Reserve, Luzon Island, Philippines (Castillo et al. 2018).

The study was conducted in the Molawin-Dampalit 2-hectare permanent plot (Fig. 1) which is a long-term monitoring plot established within the Mt. Makiling Forest Reserve, Luzon, Philippines (14°08'14'' N and 121°11'33'' E) (Tumaneng and Galang 2012) by the Makiling Center for Mountain Ecosystems and College of Forestry and Natural Resources. The study site lies within the Molawin - Dampalit subwatershed located 350 m above sea level and 65 km south of Metro Manila, Luzon. The subwatershed encompasses forest and agro-forest areas, the Molawin side being a natural secondary growth lowland evergreen rainforest and the Dampalit side being an agro-forest with diverse crops (Gamboa-Lapitan et al. 2011). In particular, the permanent plot is dominated mainly by balobo (*Diplodiscus paniculatus* Turcz), magabuyo (*Celtis luzonica* Warb) and pinanga (*Pinanga insignis* Becc) (Castillo et al. 2018). The majority of trees have 10-20 cm diameter at breast height (DBH), although remarkable trees with 120-150 cm DBH are also present. Average tree height is 10-15 m with few individuals reaching 30-35 m. The area is categorised under Climate Type 1, with an average annual rainfall of 2,397 mm and mean temperature from 25.9°C to 29.3°C. For easier facilitation and undertaking of field sampling, the permanent plot was divided into 4 quadrats.

### Methods

The vertebrate fauna inventory was primarily conducted using a combination of innovative and traditional methods used in biodiversity assessment (low-tech and hi-tech canopy methods), including the use of cage traps and mist-nets deployed at three different forest strata of the Molawin-Dampalit Permanent Plot. This plot was selected by the Forest CANOPI programme to house several temporary canopy towers made of bamboo and steel scaffolding (high-tech canopy access method) needed to facilitate access to the upper vertical strata of the plot’s tropical lowland evergreen rainforest. The programme deployed horizontal-vertical trapping arrays (SkyTrap HV) around the erected towers to readily access the traps and mist-nets hoisted into the sub-canopy and canopy layers. Sampling was supported with low-tech canopy rope-access methods, such as the use of standard safety gear for tree climbing following the single-rope technique (SRT), including ropes, harnesses, helmets and other rigging gear to ascend and descend from key emergent trees or canopy towers. These sampling methods were supplemented by other methods focused on a target group, such as the use of harp traps for capturing insectivorous bats, as they use echolocation to forage and are able to avoid mist-nets. We also employed purposive sampling (reach and grab) of more cryptic amphibians and reptiles. During each climb, but after servicing the traps and nets, direct searches within each stratum were done, particularly in known microhabitats, such as rock crevices, under stumps, tree cavities, leaf axils, mossy clumps and tangled roots. Given the limited height range of the understorey layer, protocols for setting up trapping and mist-netting arrays were safely done at ground level assisted with poles and ladders. Three sampling periods were undertaken from 2016 to 2018, with one sampling period for each of the forest stratum. Sampling at the lower understorey layer was conducted in July 2016, while sampling at the sub-canopy layer was conducted from March to April 2017. Sampling at the canopy layer was completed from February to March 2018. A variety of logistical problems were deemed limiting to the sampling of the emergent layer, as both the low-tech rope-access method and the high-tech use of scaffolding was insufficient to access the overstorey. Delayed construction of a high-tech tower with walkways limited our access within the 2-hectare permanent plot only to areas where temporary towers were built and rope-access could be safely rigged. These issues appear to be common across other canopy-based research worldwide and until a safer permanent canopy research facility is built on the plot can additional sampling be undertaken, particularly on the wider use of mist-nets and harp traps (Barker and Pinard 2001).

#### Cage Trapping

Small and medium-sized non-volant mammals were sampled using a variety of cage traps (mostly 4 × 11 inches in size) deployed across the understorey layer, approximately 1.5 to 2 m above the ground, mostly over fallen logs, root buttresses, tree stumps, lianas and rock mounds. The traps were baited with roasted coconut meat coated with peanut butter or ripe bananas and were placed along possible runways. The traps were checked every morning and re-baited in the afternoon. Arboreal non-volant mammals were sampled using cage traps of two sizes (small, 4 × 11 inches and large, 8 × 18 inches). The traps were tied onto tree branches which were possible pathways for rodents moving from one tree to another. Despite the difficulty in setting traps in the upper strata, a considerable number were deployed using ropes and wires to secure the cage traps onto tree trunks, slanted boles, main branches, epiphytes and lianas in the canopy and sub-canopy. Access to the upper strata was based on areas where towers had been erected or where tree climbing via SRT and free-climbing could be safely done. Traps were checked in the morning and afternoon, but were re-baited two to three days later. This was done to minimise human disturbance that might deter the normal movement of the animals. Total trap nights were uneven for the understorey, sub-canopy and canopy layers with 285, 521 and 467 traps deployed, respectively.

#### Mist-netting

Nylon mist-nets were used to capture volant mammals (bats) and birds during the sampling periods, using a standard 35mm mesh size with two different lengths of 6 m and 12 m. These were spread across existing trails and ridgetops or hoisted higher with the support of bamboo and wooden poles and attached to a rope-and-pulley rig for those deployed in the two upper strata. For the understorey sampling, nets were set from the ground up, with the top rung not more than 5 m from the forest floor. For the canopy and sub-canopy layers, mist-nets were set on large trees within the quadrats of the 2-hectare plot, the mist-nets being easily accessible except over uneven ridges and steep slopes. Mainly hoisted up with rope, mist-nets were set with heights ranging from 10 to 25 m, often placed between large trees where vertical space was least cluttered. Being set within a Protected Area, clearing of vegetation in any form to set-up mist-nets was not allowed and measures were undertaken to minimise the impact of human disturbance within the plot. Nets were checked every morning and evening for captures. The total net-nights for the understorey, sub-canopy and canopy sampling were 96, 180 and 135, respectively. This uneven deployment of mist-nets was due to the limited availability of clearances on the use of the mist-nets per stratum.

Identification of species were based on morphological characteristics as described in Alcala and Brown 1998 for amphibians, Alcala 1986 for reptiles, Kennedy et al. 2000 for birds and, for mammals Ingle and Heaney 1992, Heaney et al. 1998 and Heaney et al. 2010. Systematic Orders were based on Diesmos et al. 2015 for amphibians, Uetz et al. 2018 for reptiles, del Hoyo et al. 2018 for birds and Heaney et al. 2010 for mammals.

## Results and Discussion

**Figure 2.**
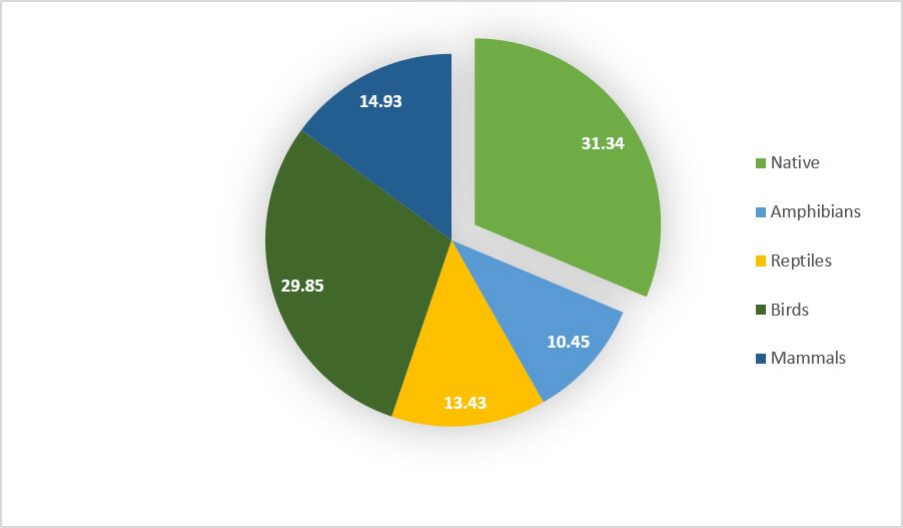
Percent endemism of recorded species.

**Figure 3.**
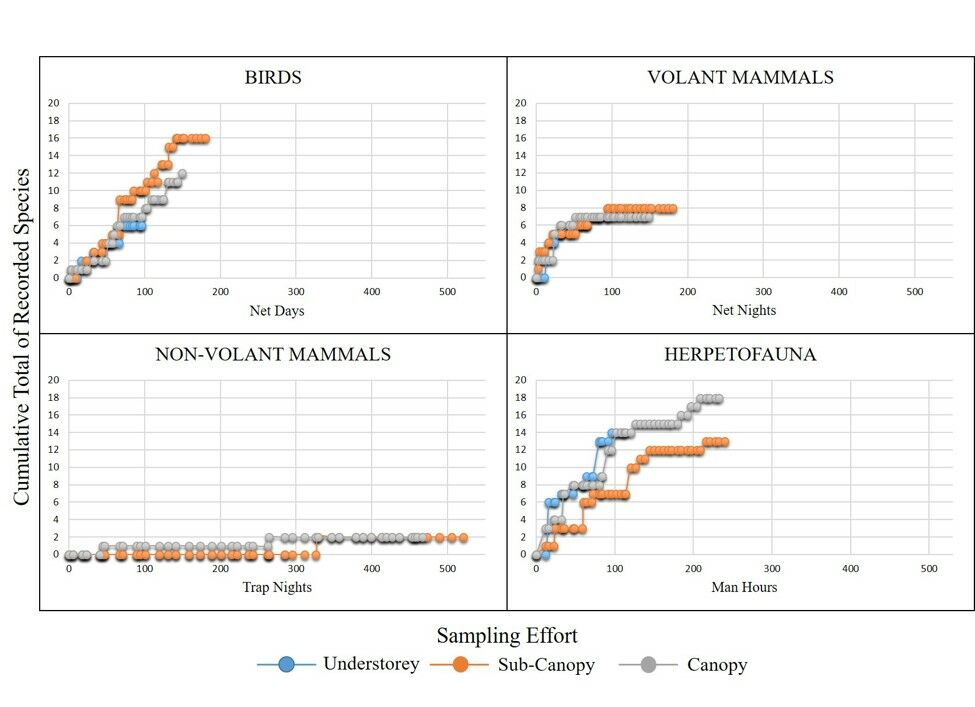
Species accumulation curves for vertebrate fauna surveyed at the Molawin-Dampalit Permanent Plot, Mt. Makiling Forest Reserve.

A total of 68 wildlife species consisting of 11 species of frogs, 15 species of reptiles (3 gekkos, 6 skinks, 2 agamids and 4 snakes), 25 species of birds and 17 species of mammals (13 bats, 3 rodents and 1 carnivore) were recorded. The majority (~69%) of the recorded species were Philippine endemics associated with forested areas (Fig. 2 and Table 1). The highest percentage of Philippine endemics was observed in birds and lowest was in mammals. Species effort curves (Fig. 3) show that sampling efforts were sufficient to record the species within the area despite the differences in the duration of sampling per forest stratum.

**Herpetofauna**

A total of 26 species was recorded, represented by 11 species of amphibians and 15 species of reptiles. Amphibians were encountered within all of the forest strata (Table 2). Of the 11 species of frogs, two species (*Platymantis luzonensis* and *Kaloula kalingensis*) were recorded in all forest strata. The most abundant in the understorey was the Common forest frog (*Platymantis dorsalis*), while in the sub-canopy and canopy, the Luzon forest frog (*Platymantis luzonensis*) was the most abundant. The majority (63%) of the recorded frog species are Philippine endemics.

**Figure 4.**
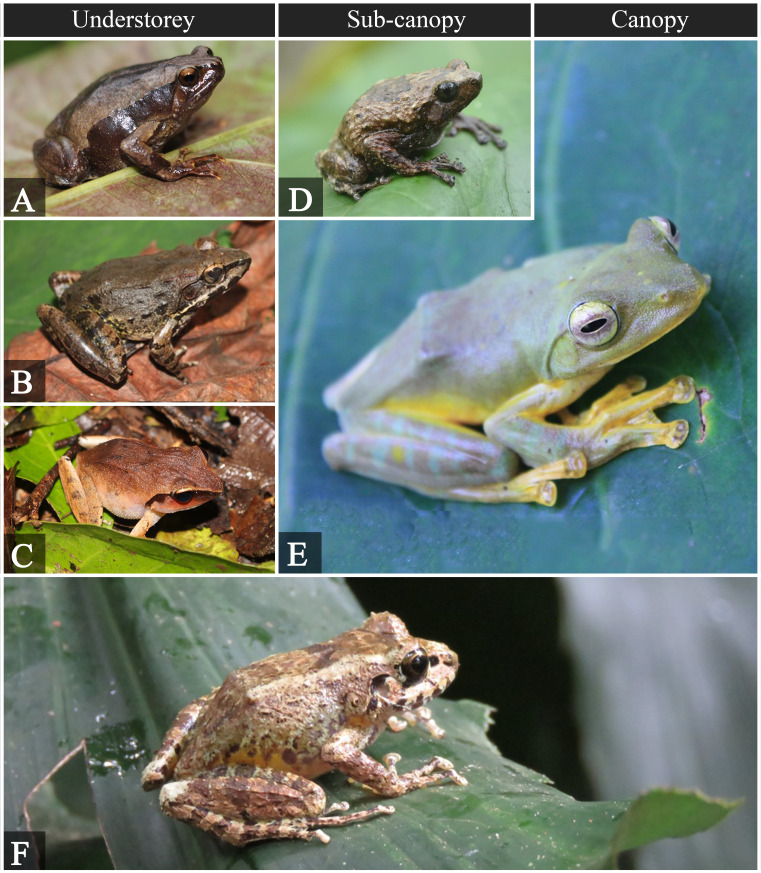
Amphibians observed at different forest strata. **A.** *Kaloula conjuncta*; **B.** *Limnonectes woodworthi*; **C.** *Platymantis corrugatus*; **D.** *Kaloula kalingensis*; **E.** *Rhacophorus pardalis*; **F.** *Platymantis luzonensis*.

Based on known reproductive modes of the four recorded *Platymantis* species, they undergo direct development which skips the tadpole stage and change into froglets immediately. They inhabit the wet forest floor and arboreal sites in closed canopy forests (Alcala and Brown 1998). Woodworth’s frog (*Limnonectes woodworthi*) and the Puddle frog (*Occidozyga laevis*) inhabit pools and banks of mountain streams, although the Puddle frog may also be found in disturbed areas with polluted waters. The two species of Chorus frogs are also forest species that burrow under the soil or climb vegetation. Both Rhacophorid frogs are foam nest builders, however, *Rhacophorus pardalis* is commonly found in the canopies of primary and secondary rainforests, while *Polypedates luecomystax* is very common and can be found in almost every habitat from forest to agricultural lands (Kuraishi et al. 2013, Manthey and Grossmann 1997). Fig. 4 shows the recorded anuran species from the understorey to the canopy.

Two new records for Mt. Makiling are the Truncate-toed chorus frog (*Kaloula conjuncta*) and Frilled tree frog (*Kurixalus appendiculatus*). *K. conjuncta* was recorded only in the understorey, while *K. appendiculatus* was only recorded in the canopy. The addition of these two new records with the previous 23 recorded frog species (Alcala et al. 1997, Sajise et al. 2005, Abraham et al. 2010, Gonzalez and Dans 1997) now brings the total species number of amphibians in MMFR to 25. The results of the sampling represent approximately 49% of the previously-recorded frog species.

A total of 15 species of reptiles was recorded in all the forest levels. Of this, 60% are Philippine endemics. Seven species were recorded in the understorey, nine species were recorded in the sub-canopy and nine species were recorded in the canopy. Three species (*Pinoyscincus jagori*, *Ahaetulla prasina* and *Cyrtodactylus philippinicus*) were recorded in all levels (Table 3). The most abundant in all levels was *Cyrtodactylus philippinicus*.

**Figure 5.**
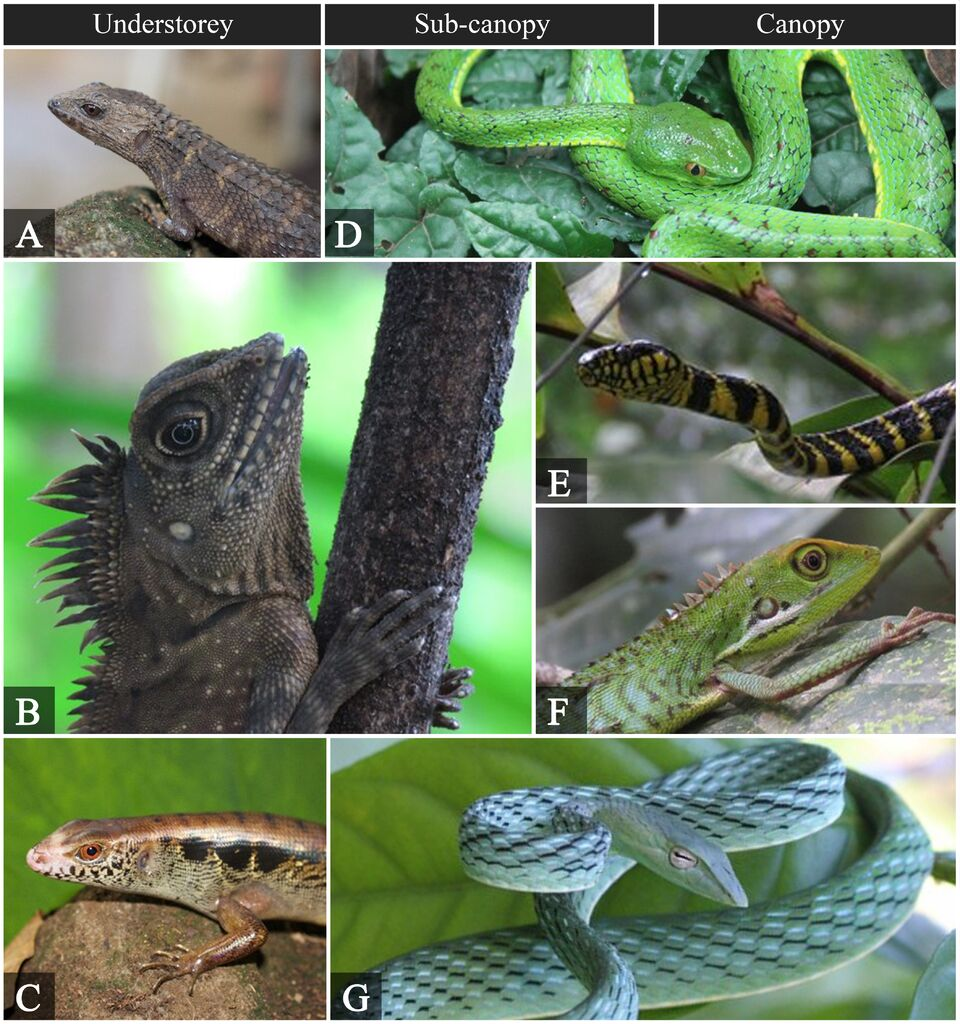
Reptiles observed at different forest strata. **A.** *Tropidophorus grayi*; **B.** *Gonocephalus sophiae*; **C.** *Otosaurus cumingi*; **D.** *Trimeresurus flavomaculatus*; **E.** *Boiga dendrophila*; **F.** *Bronchocela cristatella*; **G.** *Ahaetulla prasina*.

Three species of lizards recorded (*C. philippinicus, Gekko mindorensis* and *Psuedogekko compressicorpus*) are arboreal species. Skinks recorded are mostly ground dwellers, except for *Dasia grisea*, *Lamprolepis smaragdina* and *P. jagori* which were recorded in the sub-canopy. However, the arboreality of *P. jagori* in the sub-canopy to canopy level needs to be verified. Based on known literature, the species is frequently found in leaf litter, rotting logs and along streambeds. Its presence in the sub-canopy might be due to other factors, such as the presence of a threat or prey. The two agamids and three snakes recorded are arboreal, but hunt prey on the ground which explains their presence in the understorey. Fig. 5 shows representative species found within the permanent plot.

New records for Mt. Makiling are the Mindoro narrow-disked gekko (*Gekko mindorensis*), Northern keeled-scaled tree skink (*Dasia grisea*), Many-keeled Mabuya (*Eutropis borealis*), Negros forest dragon (*Gonocephalus sophiae*) and Smooth-scaled rat snake (*Ptyas luzonensis*). *G. mindorensis*, *E. multicarinata* and *P. luzonensis* were only recorded in the canopy, while *D. grisea* was only recorded in the sub-canopy. *G. sophiae* was recorded from the understorey to sub-canopy.

A total of 30 reptile species have been previously recorded in Mt. Makiling (Abraham et al. 2010, Gonzalez and Dans 1994, Sajise et al. 2005). The five new records thus bring the total number of recorded reptile species in MMFR to 35. The results of the survey represent 50% of the previously-recorded species in Mt. Makiling.

**Avifauna**

Twenty-five species of birds represented by 89 individuals (Table 4) were captured through netting. We are aware that this is an underestimate since sampling was limited only to mist-netting to record birds in a particular forest stratum. However, this is a more accurate assessment of vertical habitat use as birds have the ability to fly between layers. Thus, in order to make the results for birds comparable with amphibians, reptiles and mammals, we only used the data sampled through netting in this analysis. Nonetheless, other methods (ex. point counts) were used to record birds across the strata during the Forest CANOPI Program, but results were not included in this paper. Most (> 80%) were endemic and forest obligates, ranging from frugivores to insectivores and carnivores. Six species were recorded in the understorey. However, a number of species possibly present within the area are ground-dwelling birds such as junglefowl, quails, rails and others which were not recorded. This may be due to the disturbances within the permanent plot at the time of sampling as a result of various on-going research projects, visitors to the reserve and proximity to human settlements (with their free-roaming dogs, cats and chickens). There were 16 species within the sub-canopy, while 13 species were recorded within the canopy.

**Figure 6.**
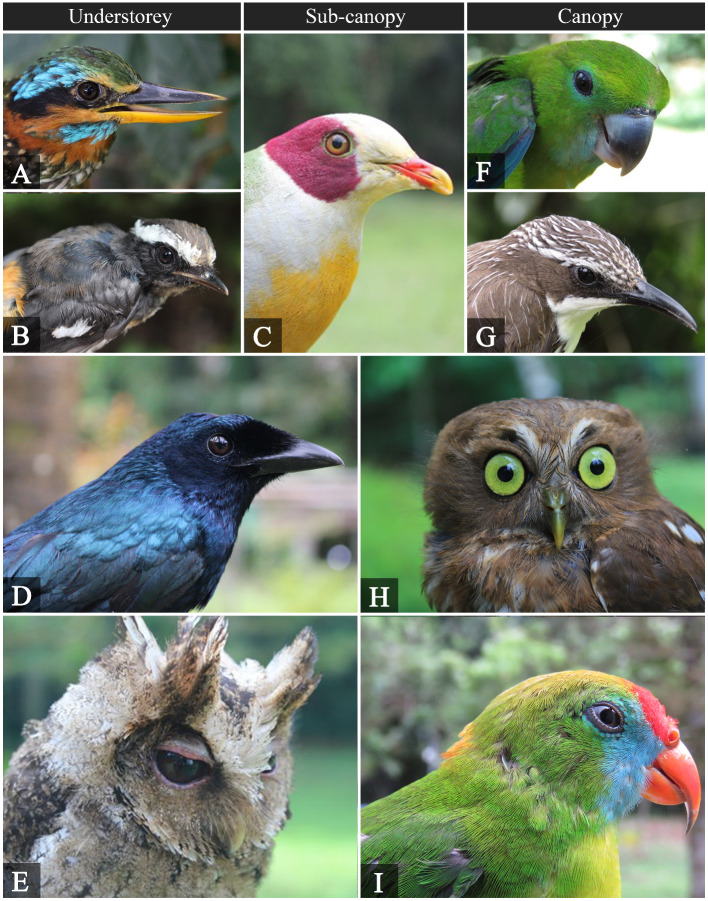
Birds observed at different forest strata. **A.** *Actenoides lindsayi*; **B.** *Copsychus luzoniensis*; **C.** *Ptilinopus occipitalis*; **D.** *Dicrurus balicassius*; **E.** *Otus megalotis*; **F.** *Bolbopsittacus lunulatus*; **G.** *Rhabdornis mystacalis*; **H.** *Ninox philippensis*; **I.** *Loriculus philippensis*.

Amongst the 25 bird species, none was common to all forest strata. Overlaps were observed between the understorey and the sub-canopy and between the sub-canopy and the canopy. Between the understorey and the sub-canopy, four species (*Dicrurus balicassius*, *Pycnonotus urostictus*, *Actenoides lindsayi* and *Otus megalotis)* were observed. Meanwhile, between the sub-canopy and the canopy, five species (*Ninox philippensis*, *Loriculus philippensis*, *Bolbopsittacus lunulatus*, *Sarcops calvus* and *Phylloscopus cebuensis*) were recorded. Fig. 6 shows representative bird species amongst the forest strata.

A total of 215 bird species (Abraham et al. 2010, de Guia et al. 2011, Gonzalez and Dans 1997, Sajise et al. 2005) had been previously recorded in Mt. Makiling. Our results represent only 11% of the species numbers recorded in previous studies. Again, this is expected to be an underestimate resulting from the focused methodology which relied only on netting.

**Mammals**

A total of 17 species consisting of 13 species of bats (Table 5) and four species of non-volant mammals were recorded. There was approximately 59% endemicity with all species associated with forested areas, albeit with varying degrees of tolerance to disturbances.

**Figure 7.**
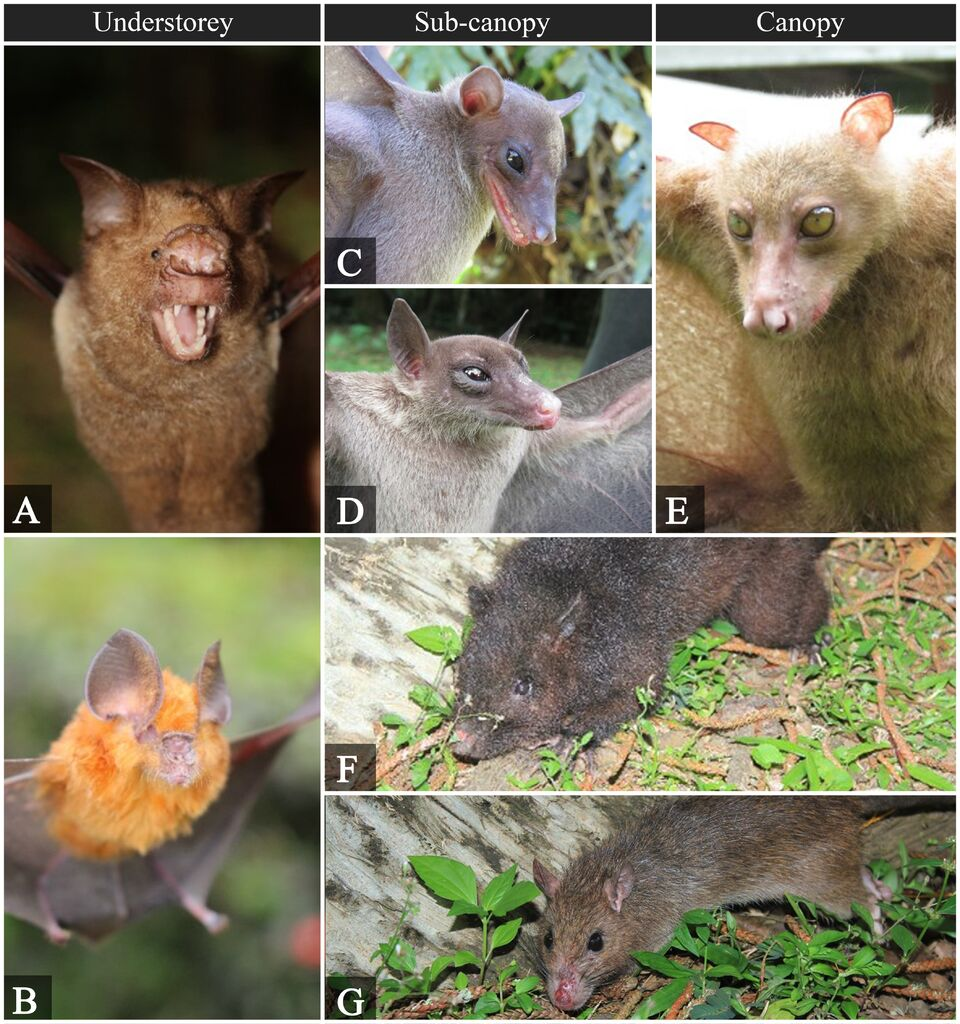
Mammals observed at different forest strata. **A.** *Hipposideros diadema*; **B.** *Hipposideros antricola*; **C.** *Rousettus amplexicaudatus*; **D.** *Eonycteris robusta*; **E.** *Desmalopex leucopterus*; **F.** *Phloeomys cumingi*; **G.** *Rattus everetti*.

For the understorey survey, only six species of bats were recorded. Two were frugivorous and the rest were insectivorous. For the sub-canopy sampling, six species of pteropodid bats and two species of insectivorous bats plus two rodents were recorded. For the canopy sampling, seven species of fruit bats, three rodents and a civet were recorded. Only two species were common to all levels (*Cynopterus brachyotis* and *Ptenochirus jagori*). Fig. 7 shows some of the recorded mammal species in the permanent plot.

New records for Mt. Makiling are the Philippine dusky roundleaf bat (*Hipposideros antricola*) and the Philippine dawn bat (*Eonycteris robusta*). *H. antricola* was only recorded in the understorey, while *E. robusta* was only recorded in the canopy. A total of 50 mammal species (Abraham et al. 2010, de Guia et al. 2011, Gonzalez and Dans 1997, Heaney et al. 1998) have been reported in Mt. Makiling. Our two new records now bring the total to 52 species. The results of our study represents ~34% of the total species recorded. This underlies the potential for more species records or even new species discovery with higher forest strata samplings.

**Trends in species richness, based on forest vertical stratification**

**Figure 8.**
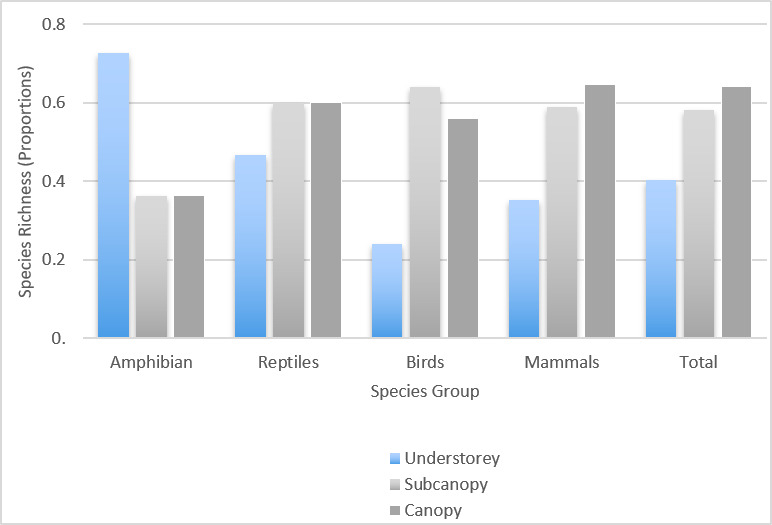
Comparison of species richness amongst forest strata for each terrestrial vertebrate group.

To compare the different fauna found between different vertical forest stratification, species-richness values were compared using proportions. Overall, our results showed that species richness increased from the understorey to the canopy layer (Fig. 8). The similarity between strata were computed using the Sørensen similarity index (θ) (Table 6). Highest similarity was observed between the sub-canopy and the canopy (θ_subxcan_ = 0.56) indicating an overlap between the species that occupy these two strata. Lowest similarity was observed between the canopy and the understorey (θ_underxcan_= 0.19) suggesting exclusivity of species between the two forest strata. This is also the case between the understorey and the sub-canopy (θ_underxsub_= 0.37), although not as low as between the understorey and the canopy. Due to increasing forest heterogeneity brought about by greater vertical stratification, species richness and composition was high in the upper strata, as this affords more vertical habitat use, in particular, canopy use (Vieira and Monteiro-Filho 2003).

Species richness of a vertebrate group varied amongst the forest strata. It has been generally observed that, except for amphibians, all other vertebrate taxa have increasing species richness the higher the vertical stratification. Scheffers et al. (2013) stated that the arboreality of frogs decreases at lower elevations and increases at higher elevations specifically from 900 m a.s.l. and above. Based on the data gathered during the sampling, the understorey had a species richness of 0.7 and sub-canopy and canopy with 0.36, coinciding with the low elevation of the study site at 350 m a.s.l. Furthermore, in terms of amphibian diversity, the understorey had low similarity with the sub-canopy and the canopy (θ_frogs_ = 0.33; 0.18)

Increasing species richness with higher vertical forest strata was observed for reptiles (understorey = 0.46; sub-canopy = 0.6; canopy = 0.6). The similarity between the understorey and the sub-canopy and canopy was also low (θ_reptiles_ = 0.44; 0.43) Trends in reptilian species richness may also be highly affected by the high complexity and heterogeneity of habitats (Vignoli et al. 2017). As an example in Vitt and Caldwell (2013), reptile species richness was influenced by horizontal stratification caused by habitat patchwork or fragmentation rather than vertical stratification. Our results suggest that species richness is influenced by vertical stratification and may be due to the presence of rich canopies with branches filled with epiphytes and various vines.

For birds, Jayson and Matthew (2003) determined that higher bird species richness was found in the middle strata compared to the lower and top strata. This concurs with our results as species richness was also highest in the sub-canopy (u = 0.24; s= 0.64 and u = 0.56). Higher foliage density was observed within the sub-canopy which may be utilised by birds for feeding and nesting. Similarities amongst the three strata were also quite low (θ_underxcan_ = 0.19; θ_subxcan =_ 0.39; θ_underxsub_ = 0.34) although the sub-canopy and the canopy are more similar.

For bats, Carvalho et al. (2013) observed that bat diversity increases with vertical stratification. Overall, this was also our observation (u = 0.35; s = 0.58; and c=0.65). However, more insectivorous bats were captured in the understorey, while more frugivorous bats were captured in the sub-canopy. Insectivorous bat activity across three vertical strata in south-eastern Australian forests were influenced by vegetation structure. Mean bat activity was > 10x higher in the sub-canopy and canopy than in the understorey, whereas bat activity was lower in the two upper strata of young regrowth forests than in old regrowth, due to more cluttered vegetation and less vertical space available. Less cluttered vegetation in forest strata offer more vertical space and this higher index of vegetation openness influences greater bat activity (Adams et al. 2009). For non-volant mammals, the species richness is greatly affected by forest structural complexity (Grelle 2003). This may explain the absence of non-volant mammals in the understorey during the sampling. The same similarity patterns were also observed with mammals. Highest similarity was observed between the sub-canopy and the canopy while the least similarity was observed between the understorey and the canopy (θ_underxcan_ = 0.24; θ_subxcan =_ 0.67; θ_underxsub_ = 0.38). Arboreal species of small mammals were observed to exclusively inhabit the upper forest strata (canopy and sub-canopy) of Atlantic rainforests in southeast Brazil and, being adapted to canopy use, they can no longer compete for habitats with other small mammals in the forest floor (Vieira and Monteiro-Filho 2003).

### Research Implications

Amongst the tropical regions, southeast Asia is a major hub of wildlife trade and also has the fastest rate of deforestation and forest conversion which could lead to rapid loss of biodiversity (Sodhi et al. 2004). Rainforest losses indicate that the potential research areas for canopy science are also at risk of disappearing. The tropical lowland evergreen rainforest is one of the key forest habitat types described by Fernando et al. (2008), characterised by tall trees, large volume of biomass, high rainfall and rich species diversity. Those with prominent dipterocarps are most extensive in southeast Asia and Malesia up to 1,500 m elevation. The dense canopy and well-developed multi-tiered stratification features emergent evergreens that rise up to 70-80 m (Yamada 1997, Poffenberger 1999).

Results of our study suggest that forest stratification in a lowland evergreen rainforest promotes wildlife diversity probably by reducing competition and promoting resource partitioning and vertical segregation. This will hopefully encourage further studies on how patterns of vertical stratification and canopy use is important in evaluating mechanisms that influence wildlife species diversity and composition in forest communities (Vieira and Monteiro-Filho 2003) including factors, such as varying environmental conditions amongst the forest layers (Acevedo and Ataroff 2012).

Some 15% of global tropical forests occur in southeast Asia and Malesia. The total forest cover in this region was estimated at 268M ha in 1990, but had dropped to 236M ha after only two decades due to conversion to plantations, logging, mining and fires (Stibig et al. 2014). A large percentage of the studies done on rainforests are limited to the ground level where access is greatest, but this only accounts for some 20–25% of the actual information. Forest canopies contain a major portion of the global biodiversity of organisms and constitute the bulk of photosynthetically-active foliage and forest biomass, especially in tropical ecosystems (Lowman and Wittman 1996). They serve as one of the drivers of biochemical processes in forest ecosystems. Thus, canopy research is essential in improving our understanding of forest ecology (Nakamura et al. 2017) and the fauna that inhabit them.

## Conclusion

Our study showed that, for most of the vertebrate fauna, species richness increases from the understorey to the canopy levels of a lowland evergreen rainforest. It follows the hypothesis that distinct stratification and increasing vertical complexity contribute to upper vertical habitat utilisation and, therefore, more species are partitioned or segregated in each stratum. As a result, the increased forest heterogeneity afforded by vertical stratification not only maintains species richness, but also increases in the upper strata, due to more canopy use. The disparity of records of vertebrate fauna between the more accessible understorey versus the less-studied canopy level, as well as other upper strata of the complex lowland evergreen rainforest indicates that many ground-based studies under-assess the species present. This has implications with the standard methods limited to understorey sampling which underestimates the species that are present in forested areas. Thus, it is imperative to include the sub-canopy and the canopy layers during sampling to obtain a more accurate inventory of species essential in designing conservation strategies for wildlife. More passive observation methods are also recommended for further studies to reduce disturbances and possible stress to the animals.

## Author Contributions

JCTG, APOdG, JCD and NCB conceived the research project. WOG and KVP conducted the fieldwork and wrote the draft reports under the supervision of JCTG, APOdG and JCD. APOdG wrote the first draft of the manuscript. All authors read and edited the manuscript.

## Figures and Tables

**Table 1.** Number of species per forest stratum and corresponding percent endemism.

**Species Group**	**Understorey**		**Sub-canopy**		**Canopy**		**Overall**	
	**No. of Species**	**Percent Endemism (%)**	**No. of Species**	**Percent Endemism (%)**	**No. of Species**	**Percent Endemism (%)**	**No. of Species**	**Percent Endemism (%)**
**Amphibians**	8	87.5	4	50	4	50	11	63.6
**Reptiles**	7	71.4	9	77.8	9	66.7	15	60
**Birds**	6	100	16	87.5	14	69.2	25	80
**Mammals**	6	50	10	70	11	54.5	17	58.8
**Total**	27	77.8	39	76.9	43	60.5	68	68.65

**Table 2.** List of recorded frog species.

**Taxa**	**Common Name**	**Residency Status**	**Habitat Association**	**Feeding Guild**	**Vertical Strata**		
					**Understorey**	**Sub-canopy**	**Canopy**
**Class Amphibia**							
**Order Anura**							
**Family Ceratobatrachidae**							
*Platymantis dorsalis* (Duméril, 1853)	Common forest frog	Philippine Endemic	Forest	Insectivore	+		
*Platymantis corrugatus* (Duméril, 1853)	Rough-backed forest frog	Philippine Endemic	Forest	Insectivore	+		
*Platymantis mimulus* (Brown, Alcala and Diesmos, 1997)	Diminutive forest frog	Luzon Endemic	Forest	Insectivore	+		
*Platymantis luzonensis* (Brown, Alcala, Diesmos and Alcala, 1997)	Luzon forest frog	Luzon Endemic	Forest	Insectivore	+	+	+
**Family Dicroglossidae**							
*Limnonectes woodworthi* (Taylor, 1923)	Woodworth's frog	Philippine Endemic	Forest	Insectivore	+		
*Occidozyga laevis* (Günther, 1858)	Puddle frog	Native	Forest; Open areas	Insectivore	+		
**Family Microhylidae**							
*Kaloula conjuncta* (Peters, 1863)	Truncate-Toed chorus frog	Philippine Endemic	Lowland Forest and Open areas	Insectivore	+		
*Kaloula kalingensis* (Taylor, 1922)	Kalinga narrow-mouth frog	Luzon Endemic	Forest	Insectivore	+	+	+
**Family Rhacophoridae**							
*Rhacophorus pardalis* (Günther, 1859)	Harlequin tree frog	Native	Forest	Insectivore		+	+
*Kurixalus appendiculatus* (Günther, 1858)	Frilled tree frog	Native	Forest	Insectivore			+
*Polypedates leucomystax* (Günther, 1858)	Common tree frog	Native	Forest; Wetlands	Carnivore		+	

**Table 3.** List of recorded reptile species.

**Taxa**	**Common Name**	**Residency Status**	**Habitat Association**	**Feeding Guild**	**Vertical Strata**		
					**Understorey**	**Sub-canopy**	**Canopy**
**Order Reptilia**							
**Order Squamata**							
**Suborder Lacertilia**							
**Family Gekkonidae**							
*Cyrtodactylus philippinicus* (Steindachner, 1867)	Philippine Bent-toed Gecko	Philippine Endemic	Forest	Insectivore	+	+	+
*Pseudogekko compressicorpus* (Taylor, 1915)	Cylindrical-bodied smooth-scaled gecko	Philippine Endemic	Forest	Insectivore		+	+
*Gekko mindorensis* (Taylor, 1919)	Mindoro Narrow-disked Gekko	Philippine Endemic	Forest	Insectivore		+	+
**Family Scincidae**							
*Tropidophorus grayi* (Günther, 1861)	Spiny Waterside Skink	Philippine Endemic	Forest; Open areas	Insectivore	+		
*Sphenomorphus cumingi* (Gray, 1845)	Cuming's Sphenomorphus	Philippine Endemic	Forest	Insectivore	+		
*Pinoyscincus jagori* (Peters, 1864)	Jagor's Sphenomorphus	Philippine Endemic	Forest	Insectivore	+	+	+
*Dasia grisea* (Gray, 1845)	Northern Keeled-scaled tree skink	Native	Forest	Insectivore		+	
*Eutropis borealis* (Brown & Alcala, 1980)	Many-keeled Maboua	Native	Forest	Insectivore			+
*Lamprolepis smaragdina* (Lesson, 1829)	Emerald Tree Skink	Native	Forest	Insectivore		+	
**Family Agamidae**							
*Gonocephalus sophiae* (Gray, 1845)	Negros Forest Dragon	Philippine Endemic	Forest	Insectivore	+	+	
*Bronchocela cristatella* (Kuhl, 1820)	Green Crested Lizards	Native	Forest	Insectivore		+	+
**Suborder Ophidia**							
**Family Colubridae**							
*Boiga dendrophila* (Boie, 1827)	Mangrove Snake	Native	Forest	Carnivore	+		+
*Ahaetulla prasina* (Boie, 1827)	Asian Vine Snake	Native	Forest; Agricultural Lands	Carnivore	+	+	+
**Family Viperidae**							
*Trimeresurus flavomaculatus* (Gray, 1842)	Philippine Pit Viper	Philippine Endemic	Forest	Carnivore		+	+
**Family Colubridae**							
*Ptyas luzonensis* (Günther, 1873)	Smooth-scaled rat snake	Philippine Endemic	Forest	Carnivore		+	

**Table 4.** List of recorded bird species.

**Taxa**	**Common Name**	**Residency Status**	**Habitat Association**	**Feeding Guild**	**Vertical Strata**		
					**Understorey**	**Sub-canopy**	**Canopy**
**Order Passeriformes**							
**Family Dicruridae**							
*Dicrurus balicassius* (Linnaeus, 1766)	Balicassiao	Philippine Endemic	Forest	Insectivore	+	+	
**Family Pycnonotidae**							
*Pycnonotus urostictus* (Salvadori, 1870)	Yellow-wattled bulbul	Philippine Endemic	Forest	Omnivore	+	+	
*Hypsipetes philippinus* (Forster, 1795)	Philippine bulbul	Philippine Endemic	Forest	Omnivore		+	
**Family Phylloscopidae**							
*Phylloscopus cebuensis* (Dubois, 1900)	Lemon-throated leaf-warbler	Philippine Endemic	Forest	Insectivore		+	+
**Family Monarchidae**							
*Terpsiphone cinnamomea* (Sharpe, 1877)	Southern rufous paradise flycatcher	Philippine Endemic	Forest	Insectivore		+	
**Family Muscicapidae**							
*Copsychus luzoniensis* (Kittlitz, 1832)	White-browed shama	Philippine Endemic	Forest	Insectivore	+		
**Family Rhipiduridae**							
*Rhipidura cyaniceps* (Cassin, 1855)	Blue-headed fantail	Philippine Endemic	Forest	Insectivore	+		
**Family Dicaeidae**							
*Dicaeum hypoleucum* (Sharpe, 1876)	Buzzing flowerpecker	Philippine Endemic		Frugivore		+	
**Family Nectariniidae**							
*Anthreptes griseigularis* (Tweeddale, 1878)	Grey-throated sunbird	Philippine Endemic	Forest	Omnivore			+
**Family Hirundinidae**							
*Hirundo tahitica* (Gmelin, 1789)	Pacific swallow	Native	Forest; Sea coast	Insectivore			+
**Family Sturnidae**			Forest to non-forest				
*Sarcops calvus* (Linnaeus, 1766)	Coleto	Philippine Endemic	Forest	Omnivore		+	+
*Rhabdornis mystacalis* (Temminck, 1825)	Stripe-headed rhabdornis	Philippine Endemic	Forest	Omnivore			+
**Order Columbiformes**							
**Family Columbiformes**							
*Ptilinopus occipitalis* (Gray, 1844)	Yellow-breasted fruit dove	Philippine Endemic	Forest	Frugivore		+	
*Ptilinopus leclancheri* (Bonaparte, 1855)	Black-chinned fruit dove	Native	Forest	Frugivore		+	
*Phapitreron amethystinus* (Bonaparte, 1855)	Amethyst brown dove	Philippine Endemic	Forest	Frugivore			+
*Phapitreron leucotis* (Temminck, 1823)	White-eared brown dove	Philippine Endemic	Forest	Frugivore		+	
**Order Accipitriformes**							
**Family Accipitridae**							
*Accipiter soloensis* (Horsfield, 1821)	Chinese sparrowhawk	Native	Forest	Carnivore		+	+
**Order Psittaciformes**							
**Family Psittacidae**							
*Bolbopsittacus lunulatus* (Scopoli, 1786)	Guaiabero	Philippine Endemic	Forest	Frugivore		+	+
*Loriculus philippensis* (Müller, 1776)	Colasisi; Philippine hanging parrot	Philippine Endemic	Forest	Herbivore		+	+
**Order Coraciiformes**							
**Family Alcedinidae**							
*Actenoides lindsayi* (Vigors, 1831)	Spotted wood kingfisher	Philippine Endemic	Forest	Carnivore	+	+	
**Order Strigiformes**							
**Family Strigidae**							
*Otus megalotis* (Walden, 1875)	Philippine Scops-Owl	Philippine Endemic	Forest	Carnivore	+	+	
*Ninox philippensis* (Bonaparte, 1855)	Luzon boobook; Philippine hawk owl	Philippine Endemic	Forest	Carnivore		+	+
**Order Bucerotiformes**							
**Family Bucerotidae**							
*Penelopides manillae* (Boddaert, 1783)	Luzon hornbill	Philippine Endemic	Forest	Frugivore			+
**Order Caprimulgiformes**							
**Family Apodidae**							
*Collocalia troglodytes* (Gray, 1845)	Pygmy swiftlet	Philippine Endemic	Forest; Inland water	Insectivore			+
*Collocalia marginata* (Salvadori, 1882)	Grey-rumped swiftlet	Philippine Endemic	Forest; Human habitats; Urban areas	Insectivore			+

**Table 5.** List of recorded mammal species.

**Taxa**	**Common Name**	**Residency Status**	**Habitat Association**	**Feeding Guild**	**Vertical Strata**		
					**Understorey**	**Sub-canopy**	**Canopy**
**Order Chiroptera**							
**Family Pteropodidae**							
*Cynopterus brachyotis* (Müller, 1838)	Common short-nosed fruit bat	Native	Forest	Frugivore	+	+	+
*Ptenochirus jagori* (Peters, 1861)	Musky fruit bat	Philippine Endemic	Forest	Frugivore	+	+	+
*Macroglossus minimus* (É. Geoffroy Saint-Hilaire, 1810)	Long-tongued nectar bat	Native	Forest	Frugivore		+	+
*Rousettus amplexicaudatus* (É. Geoffroy Saint-Hilaire, 1810)	Common rousette	Native	Forest; Caves; Agricultural Lands	Frugivore		+	+
*Eonycteris robusta* (Miller, 1913)	Philippine dawn bat	Philippine Endemic	Forest; Caves	Frugivore		+	+
*Eonycteris spelaea* (Dobson, 1871)	Common dawn bat	Native	Forest; Caves	Frugivore			+
*Haplonycteris fischeri* (Lawrence, 1939)	Philippine pygmy fruit bat	Philippine Endemic	Forest	Frugivore		+	
*Desmalopex leucopterus* (Temminck, 1853)	White-winged flying fox	Philippine Endemic	Forest	Frugivore			_+_
**Family Vespertilionidae**							
*Myotis muricola* (Gray, 1864)	Whiskered myotis	Native	Forest	Insectivore	+		
**Family Hipposideridae**							
*Hipposideros diadema* (Geoffroy, 1813)	Diadem roundleaf bat	Native	Forest; Caves	Carnivore	+	+	
*Hipposideros antricola* (Peters, 1861)	Philippine dusky roundleaf bat	Philippine Endemic	Forest; Caves	Carnivore	+		
*Hipposideros obscurus* (Peters, 1861)	Philippine forest roundleaf bat	Philippine Endemic	Forest; Caves	Insectivore	+		
**Family Rhinolophidae**							
*Rhinolophus inops* (Andersen, 1905)	Philippine forest horseshoe bat	Philippine Endemic	Forest	Insectivore		+	
**Order Rodentia**							
**Family Muridae**							
*Phloeomys cumingi* (Waterhouse, 1839)	Southern Luzon giant slender-tailed cloud rat	Philippine Endemic	Forest	Frugivore		+	+
*Rattus everetti* (Günther, 1879)	Philippine forest rat	Philippine Endemic	Forest	Omnivore		+	+
*Apomys sp*.	Philippine rat mice	Philippine Endemic	Forest	Omnivore			+
**Order Carnivora**							
**Family Viverridae**							
*Paradoxurus philippinensis* (Jourdan, 1837)	Common palm civet	Native	Forest; Agricultural areas	Omnivore			+

**Table 6.** Simpson Similarity Index between forest strata.

	Understorey vs. Sub-canopy	Sub-canopy vs. Canopy	Canopy vs. Understorey
Amphibians	0.33	0.67	0.18
Reptiles	0.44	0.7	0.43
Birds	0.36	0.39	0.19
Mammals	0.38	0.67	0.24
Total	0.37	0.56	0.19
